# Flavonoids and Isoflavonoids Biosynthesis in the Model Legume *Lotus japonicus*; Connections to Nitrogen Metabolism and Photorespiration

**DOI:** 10.3390/plants9060774

**Published:** 2020-06-20

**Authors:** Margarita García-Calderón, Carmen M. Pérez-Delgado, Peter Palove-Balang, Marco Betti, Antonio J. Márquez

**Affiliations:** 1 Departamento de Bioquímica Vegetal y Biología Molecular, Facultad de Química, Universidad de Sevilla, Calle Profesor García González, 1, 41012-Sevilla, Spain; marbioq@us.es (M.G.-C.); cmperez@us.es (C.M.P.-D.); mbetti@us.es (M.B.); 2 Institute of Biology and Ecology, Faculty of Science, P.J. Šafárik University in Košice, Mánesova 23, SK-04001 Košice, Slovakia; peter.palove-balang@upjs.sk

**Keywords:** flavonoids, isoflavonoids, phenolics, legumes

## Abstract

Phenylpropanoid metabolism represents an important metabolic pathway from which originates a wide number of secondary metabolites derived from phenylalanine or tyrosine, such as flavonoids and isoflavonoids, crucial molecules in plants implicated in a large number of biological processes. Therefore, various types of interconnection exist between different aspects of nitrogen metabolism and the biosynthesis of these compounds. For legumes, flavonoids and isoflavonoids are postulated to play pivotal roles in adaptation to their biological environments, both as defensive compounds (phytoalexins) and as chemical signals in symbiotic nitrogen fixation with rhizobia. In this paper, we summarize the recent progress made in the characterization of flavonoid and isoflavonoid biosynthetic pathways in the model legume *Lotus japonicus* (Regel) Larsen under different abiotic stress situations, such as drought, the impairment of photorespiration and UV-B irradiation. Emphasis is placed on results obtained using photorespiratory mutants deficient in glutamine synthetase. The results provide different types of evidence showing that an enhancement of isoflavonoid compared to standard flavonol metabolism frequently occurs in *Lotus* under abiotic stress conditions. The advance produced in the analysis of isoflavonoid regulatory proteins by the use of co-expression networks, particularly MYB transcription factors, is also described. The results obtained in *Lotus japonicus* plants can be also extrapolated to other cultivated legume species, such as soybean, of extraordinary agronomic importance with a high impact in feeding, oil production and human health.

## 1. Introduction

The use of nitrogen by plants involves several steps, including uptake, assimilation, translocation, and different forms of recycling and remobilization processes, all of them of crucial importance in terms of nitrogen utilization efficiency. Different processes exist in plants, which give rise to the production of endogenous sources of ammonium which have to be efficiently re-assimilated by secondary ammonium assimilation. These processes include photorespiration, the biosynthesis of phenylpropanoids, as well as ureide, nucleotide and amino acid catabolism [1]. Phenylpropanoid metabolism represents an important metabolic pathway from which originates a wide number of secondary metabolites derived from phenylalanine or tyrosine, including monolignols, flavonoids and isoflavonoids, various phenolic acids, and stilbenes [2]. It is well known that secondary metabolites are crucial molecules in plant life, as protective agents against environmental factors (e.g., oxidative stress, pathogens, etc.) as well as elements favoring reproduction [3,4,5,6]. In particular, it is well established that phenylpropanoid-derived compounds have roles in plant growth and development, and in the defense against biotic and abiotic stress [2]. The phenylpropanoid pathway has different branches that lead to different families of compounds, such as chalcones, flavones, flavonols, flavanones, isoflavonoids, and anthocyanins, among others [7]. The structure, composition and biological activity of flavonoids have been frequently analyzed (see [7,8,9,10] for an overview, and references therein).

The second most important family of crop plants for humans, after Poaceae, are Fabaceae because they provide sources of food, feed for livestock and raw materials for industry [11]. Legumes are crucial plants in sustainable agriculture because they are able to fix atmospheric dinitrogen in a symbiotic association with rhizobial species. In addition, legumes produce a high diversity of secondary metabolites which serve as defense compounds against herbivores and microbes, but also as signal compounds to attract pollinating and fruit-dispersing animals. As nitrogen-fixing organisms, legumes produce more nitrogen containing secondary metabolites than other plant families [12]. In particular, flavonoids and isoflavonoids, which are compounds lacking nitrogen in their structures, are postulated to play pivotal roles in the adaptation of legumes to their biological environments both as defensive compounds (phytoalexins) and as chemical signals in symbiotic nitrogen fixation with rhizobia [13]. A primary function of flavonoids in legume–rhizobia symbiosis is to induce transcription of the genes involved in the biosynthesis of Nod factors. These factors are rhizobial signaling molecules perceived by the plant to allow symbiotic infection of the root. Many legumes produce specific flavonoids that only induce Nod factor production in compatible rhizobia, and therefore act as important determinants of host range [14]. Despite a wealth of evidence on legume flavonoids, relatively few have proven roles in rhizobial infection. The molecular details of how flavonoid production in plants is regulated during nodulation have not yet been clarified, but nitrogen availability has been shown to play a role [15]. The role of flavonoids and isoflavonoids in plant symbiosis is not limited to nitrogen-fixing bacteria since these compounds also play several roles in the symbiosis with mutualistic fungi. During the establishment of fungal symbiosis, these compounds can stimulate spore germination, hyphal branching and growth, root colonization, and arbuscule formation inside the root [16]. In later stages of symbiosis, flavonoids may be involved in the autoregulation of mycorrhization [17]. In the case of soybean, a specific isoflavonoid rather than a flavonoid can stimulate hyphal growth [18]. These effects often are host-specific, much like in the case of plant–rhizobial symbiosis. In fact, autoregulation of nodulation and autoregulation of mycorrhizae, the two negative feedback loops that control the formation of rhizobial and mycorrhizal symbioses, may share common elements [19]. However, the inhibitory effects of some plant flavonoids on fungal symbiosis have also been reported, both in plants that are host for mycorrhizal fungi and in non-host plants ([16], and references therein). Flavonoids can also accumulate in the early stages of plant–fungi interaction as a defense response; however, once the symbiosis has been established, the fungal symbiont may use the flavonoids as carbon source [20]. In addition, because legumes are a significant source of food and forage, the effects of leguminous flavonoids and isoflavonoids on human and animal health are being studied intensively [13,21]. In particular, excellent reviews describe exhaustively the different isoflavonoids compounds found in legume plants [22,23,24]. 

A major impetus in the investigation of the phenylpropanoid pathway in forage legumes was the fact that proanthocyanidins are beneficial in the diet of grazing ruminants through reduced pasture bloat, increase protein uptake and reduced intestinal parasite burdens [25,26]. Bloat is caused by protein foam formed in the rumen when animals graze protein-rich legume pastures. Rumen foam prevents normal expulsion of gases and, as consequence, ruminal volume and intraruminal pressure increase [26]. In the presence of proanthocyanidins, excess dietary proteins as well as bacterial enzymes are complexed and the level of protein degradation in the rumen is significantly reduced. This leads to an increased protein bypass to the ruminant’s gut and the improved absorption of essential amino acids, resulting in increased milk and meat production [25,26,27,28]. The production of pasture legume species with moderate amounts of foliar proanthocyanidins (2–4%) is of considerable interest to the pastoral agricultural industry [25,28,29].

Genomics and functional genomics, together with genetics, biochemistry, physiology, and molecular and cell biology, have accelerated discoveries in legume molecular and systems biology. Unfortunately, agricultural legumes are relatively poor model systems for research in genetics and genomics. Studies on most of the major leguminous crops are hampered by large genome sizes and other disadvantages (allogamy, polyploidy, transformation or regeneration recalcitrancies, few or large seeds and seedlings, genome duplications, long generation times, etc.). As a result, two species, *Lotus japonicus* and *Medicago truncatula*, were adopted internationally as models for modern legume research [30,31] and important advances have been produced in understanding the molecular details of rhizobial–legume symbiosis [32,33,34,35,36,37]. The high levels of synteny that exist between the different legume genomes imply that the advances obtained with the model plants can be used in order to understand and improve the performance of cultivated legume species [38].

In this paper, we will summarize recent progress made in the characterization of flavonoid and isoflavonoid biosynthetic pathways in legume plants with a particular focus on the model legume *Lotus japonicus*, and the impact that these studies may have to improve cultivated legumes of great agronomic importance such as soybean (*Glycine max*). 

## 2. Flavonoid and Isoflavonoid Biosynthetic Pathways in *Lotus*

**Figure 1:**
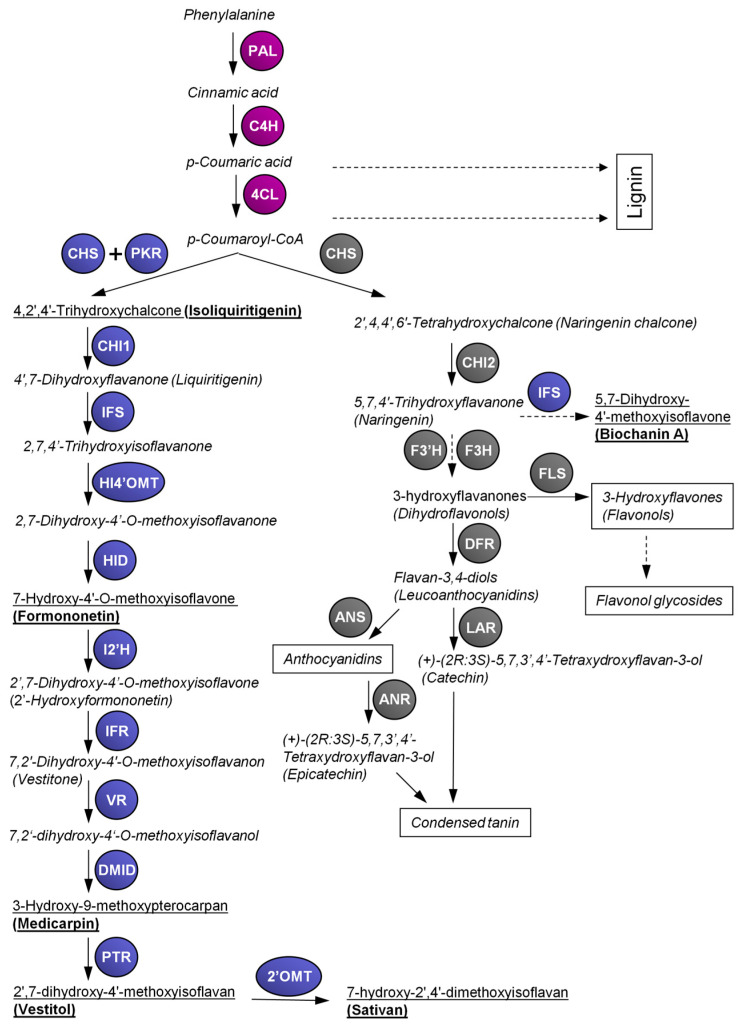
Overview of the flavonoid and isoflavonoid pathways in *Lotus japonicus*. 4CL, 4-coumarate:CoA ligase; 2’OMT, 2’-O-methyltransferase; I2’H, isoflavone-2’-hydroxylase; ANR, anthocyanidin reductase; ANS, anthocyanidin synthase; C4H, cinnamic acid 4-hydroxylase; CHI, chalcone isomerase; DMID, 7,2’-dihydroxy-4’-O-methoxyisoflavanol dehydratase (syn. pterocarpan synthase); CHS, chalcone synthase; DFR, dihydroflavonol 4-reductase; F3H, flavanone 3-hydroxylase; F3’H, flavanone 3’-hydroxylase; FLS, flavonol synthase; HID, 2-hydroxyisoflavanone dehydratase; HI4’OMT, 2-hydroxyisoflavanone 4’-*O*-methyltransferase; IFR, isoflavone reductase; IFS, 2-hydroxyisoflavanone synthase; LAR, leucoanthocyanidin reductase; PAL, phenylalanine ammonia lyase; PKR, polyketide reductase (syn. chalcone reductase); PTR, pterocarpan reductase; VR, vestitone reductase. Purple color: enzymes of general phenylpropanoid pathway; grey color: enzymes of flavonoid pathway; blue color: enzymes of isoflavonoid pathway. Dashed arrows represent multiple biosynthetic steps. Trivial names of compounds are presented if they are commonly used; the others are presented by their semi-systematic names. Semi-systematic names and chemical structures of the referred flavonoids and isoflavonoids are attached online in Table S1. The names underlined in bold highlight most abundant isoflavonoids found in *L. japonicus* according to our data [42].

The enzyme chalcone synthase (CHS) is involved in the biosynthesis of the precursor molecules for both flavonoids and isoflavonoid biosynthesis. CHS is a member of the type III polyketide synthase family that catalyzes the conjugation of three acetate units from malonyl-CoA to a *p*-coumaroyl-CoA starter molecule derived from phenylalanine via the general phenylpropanoid pathway (Figure 1). In the same active site, additional aromatic “A” cycle of flavonoids is built via the intramolecular cyclisation [39]. The product of such reaction is 2’,4,4’,6’-tetrahydroxychalcone (naringenin chalcone), later changing to 5,7,4’-trihydroxyflavanone (naringenin) by building of the “C” heterocycle catalyzed by chalcone isomerase (CHI) that serves as a precursor for the other flavonoids [40]. In some species of the family Fabaceae, isoflavonoids, such as genistein, biochanin A or others, are produced from naringenin [41]. However, most of the isoflavonoids are synthesized via isoliquiritigenin that is produced by the coupled catalytic action of CHS and chalcone reductase (CHR; also called polyketide reductase, PKR, see below). 

Whereas in *Arabidopsis thaliana* only one single gene for CHS is known, in other species several *CHS* genes were found (e.g., two in cacao, four in wild strawberry, five in apple, six in poplar), which is especially true for legumes [43,44]. In *L. japonicus* 13–14 *CHS* genes were found, 15 in *Glycine max* and 17 in *Medicago truncatula* [43]. The higher number of *CHS* genes in legumes is likely related to the presence of the isoflavonoid pathway in that family. In *L. japonicus*, *CHS6* (called *LjCHS1* in [45]) could represent the non-leguminous type of chalcone synthase; on the other hand, in soybean, *GmCHS6*, *GmCHS7* and *GmCHS8* seem more related to isoflavonoid production [46,47]. *GmCHS7* and *GmCHS8* show strong homology with *LjCHS5* (Lj1g3v2626200.1), *LjCHS8* (Lj0g3v0129339.1) *LjCHS9* (Lj2g3v2124320.1) and *LjCHS11* (Lj2g3v2124320.2), whereas *GmCHS6* is homologous to *LjCHS12* (Lj4g3v2574990.1). However, *Lotus* isoflavonoids are produced mainly via isoliquiritigenin, the daidzein and genistein (and their derivates) found in soybean are produced from isoliquiritigenin and naringenin chalcone, respectively [41,48] (Figure 1). Therefore, the regulation pattern of chalcone synthases in soybean might be more complex. 

The flavonoid biosynthetic pathway producing flavonols, anthocyanidins and proanthocyanidins (condensed tannins) in *L. japonicus* are described in Figure 1. *F3H*, *F3’H* and *FLS* genes have not been studied in detail to date—five *DFR* genes were described in a cluster on chromosome 5 by [49] and different specificities of DFR isozymes in the substrate hydroxylation patterns have been reported. The proanthocyanidins (both epicatechin and catechin type) are biosynthesized from dihydroflavonols by the action of anthocyanidin reductase (ANR) and leucoanthocyanidin 4-reductase (LAR), two gene encodings for enzymes committed to epicatechin and catechin biosynthesis, respectively, that were identified in *L. corniculatus* [50].

Higher plants share a common core flavonoid pathway, although different species frequently develop specific branches as an adaptation to diverse environmental conditions. For example, *A. thaliana* accumulates mainly flavonols (kaempferol, quercetin and isorhamnetin glycosides) in all tissues, and anthocyanidins and epicatechin types of proanthocyanidins in the seed coat under stress conditions [51]. A rising number of studies report protein–protein interactions of flavonoid biosynthetic enzymes providing evidence for weakly bound complexes called “metabolons” which are co-localized at the endoplasmic reticulum (ER) [52,53,54]. The interaction of the enzymes in the system likely allows better connection of reaction intermediates with subsequent enzymes and prevents their loss by diffusion or unfavorable cell equilibrium. Such protein–protein interactions were found for CHS, flavanone 3-hydroxylase (F3H), dihydroxyflavonol 4-reductase (DFR), anthocyanidin synthase (ANS) and also CHI or CHI-like protein (with a putative role as fatty-acid binding protein) [55], so the proposed model of metabolon comprises the enzymes necessary for formation of anthocyanidins [56]. On the other hand, there is still lack of evidence of interaction with flavanone 3’–hydroxylase (F3’H) [57]. Proanthocyanidins are produced by action of ANR, LAR and polyphenol oxidase (LAC15) resulting in the oligo-and polymers of the flavan-3-ol units. Substrate channeling between DFR and LAR was described using molecular modeling and predicted the functional significance of metabolon formation during synthesis [58]. Proanthocyanidins are produced both in shoots and roots of *Lotus* sp. However, significant differences in their accumulation may occur among different species, but also within different populations of the same species. Whereas in *L. japonicus* (and some other species) they are usually present in almost undetectable amounts, the closely related tetraploid forage species *L. corniculatus* may accumulate proanthocyanidins in considerable levels [59,60]. The highest proanthocyanidin levels were found in *L. unifoliolatus* (syn. *L. americanus*) and *L. uliginosus* (syn. *L. pedunculatus*) [59,61].

The key enzyme for flavonol formation is flavonol synthase (FLS) using dihydroflavonol substrates. *L. japonicus* is a plant that accumulates flavonol kaempferol glycosides in considerable amounts, especially kaempferol-3,7-dirhamnoside. Quercetin glycosides are present at lower levels but increase under some abiotic stress conditions [62,63]. Moreover, a considerable amount of gossypetine glycosides occurs in flowers and a small amount of isorhamnetine can be detected in stems [64]. Only the minor methylation on 3’ position of quercetine is present in *L. japonicus*, whereas the methylation at position 8 was described only in *L. corniculatus* [65], leading to presence of sexangularetin and corniculatusin in that species [66].

The production of isoliquiritigenin, the starting point of the second branch of the biosynthetic pathway, is related to the activity of CHR (also called polyketide reductase, PKR), only identified in papilionoid legumes (like *Glycine max*, *Medicago sativa*, *Glycyrrhiza echinata*, *Glycyrrhiza glabra*). Five genes and 1 pseudogene are present in the *L. japonicus* genome [35]. CHR acts in a coupled catalytic action with CHS [45]. Furthermore, two types of CHI genes are present. *LjCHI2* is highly homologous to non-legumes (also referred as type I), whereas *LjCHI1*, *LjCHI3* and *LjCHI4* are legume-specific type II, also occurring in *Medicago sativa*, *Phaseolus vulgaris*, *Pisum sativum* and *Pueraria lobata* [67] (Figure 1). The legume-specific type II evolved to produce 5-deoxy(iso)flavonoids from 6’deoxychalcone (isoliquiritigenin) along with the establishment of the Fabaceae. 

The protein–protein interaction of key enzymes of isoflavonoid pathway (CHS, CHR, CHI and IFS) that are associated with ER via cytochrome P450 has been recently demonstrated in soybean [68] as well as with the three enzymes of general phenylpropanoid pathway (PAL, C4H, 4CL) and with the last enzyme of the shikimate pathway, arogenate dehydratase (ADT), the enzyme converting arogenate to phenylalanine [69]. The enzyme complex may be associated with the ER membrane at the plastid-associated membrane sites, allowing the flux of intermediates from shikimate pathway occurring in plastids toward daidzein or glycitein isoflavones present in soybean [69,70].

Isoflavone synthase (IFS; 2-hydroxyisoflavanone synthase) is a membrane-associated enzyme belonging to the CYP93C subfamily of cytochrome P450 monooxygenases that constructs the isoflavonoid skeleton from 4’,7-dihydroxyflavanone substrate (liquiritigenin) by an unusual aryl migration reaction. At a lower rate, IFS may convert naringenin in several legume species, such as soybean [41]. IFS has been identified almost exclusively in legumes, with *Beta vulgaris* being the only known exception [71,72]. Among 273 putative P450 genes in *A. thaliana* genome, none of them has isoflavone synthesizing activity [73]. At least two functional genes of IFS (*IFS1* and *IFS2*) and one pseudogene are present in the *L. japonicus* genome [45]. *L. japonicus* IFS likely has a strong preference for liquiritigenin, although a small amount of biochanin A detected in plants on UV-B irradiation suggests a possibility of a minor activity using also naringenin as a substrate [42].

The substrate specificity of 2-hydroxyisoflavanone dehydratase (HID) may differ among species. In soybean, HID accepts 2,5,7,4’-tetrahydroxyisoflavanone or 2,7,4’-trihydroxyisoflavanone as substrate, which is then de-hydrated to produce a double bond between C-2 and C-3, yielding genistein or daidzein [23,74]. The overexpression of HID from soybean with broad substrate specificity in *L. japonicus* resulted in the production of considerable amounts of daidzein or genistein [75]. The biosynthesis of the main isoflavonoid, vestitol, in *L. japonicus* was proposed by [45], according the previous data described in *Glycyrrhiza echinata* [74]. Firstly the 4’-O-methyltransferase (HI4‘OMT) reaction occurs, and subsequent dehydration by HID yields formononetin (Figure 1), the central biosynthetic intermediate for the production of diverse isoflavonoid phytoalexins (e.g., maackiain, pisatin, medicarpin, etc.) in a number of legume species, including agronomically important ones such as pea (*Pisum sativum*) or chickpea (*Cicer arietinum*) [76]. 

In *L. japonicus*, formononetin is then converted by isoflavone-2’-hydroxylase (I2’H) to 2’,7-dihydroxy-4’-O-methoxyisoflavone and subsequently to vestitone by isoflavone reductase (IFR). The next step is the NADPH-dependent reduction of vestitone to 7,2’-dihydroxy-4’-O-methoxyisoflavanol, catalyzed by the vestitone reductase (VR) that is stereospecific for the (*3S*)-vestitone [77]. *HI4’OMT*, *HID* and *I2’H* were suggested to occur in single copies in the *L. japonicus* genome [78], but recently, more putative copies could be predicted at least in the case of *HID* (miyakoguza.jp 3.0). The putative *L. japonicus IFR1* and *VR1*, *VR2*, *VR3* and *VR4* genes (four *VR* genes) for vestitol accumulation were identified by sequence similarity with *Medicago sativa* [45]. Although their functional validation is still lacking, these genes are markedly upregulated after glutathione treatment [79]. The production of medicarpin from 7,2’-dihydroxy-4’-O-methoxyisoflavanol is catalyzed by pterocarpan synthase (PTS) that was found in *L. japonicus*, *Glycine max* and *Glycyrrhiza echinata*. This enzyme has similar biochemical properties as previously reported DMI-dehydratase in *Cicer arientinum*, *G. max* and *Medicago sativa*. This raises the question of whether the product of the *LjPTS1* gene corresponds to the enzyme described above, but the evidence available at present is not conclusive [80]. The synthesis of vestitol is then catalyzed by pterocarpan reductase (PTR). Four genes were found to encode PTR, from which *PTR3* was found to be inducible by glutathione [45]. However, *PTR1* and *PTR2* have much higher activity and enantiospecifity with (-)-medicarpin; therefore, they are considered to be responsible for vestitol production [81]. In stress conditions like UV-B application or glutathione treatment, a remarkable accumulation of sativan was observed in *L. japonicus* and *L. corniculatus* [42,82]. Production of sativan requires the activity of a 2’-O-methyltransferase to convert vestitol to sativan. Among the type I O-methyltransferases isolated from *Medicago truncatula*, *MtOMT2*, *MtOMT4*, *MtOMT5*, *MtOMT6* and *MtOMT7* showed some vestitol methylation activity, but with a very low efficiency. Furthermore, they appeared to methylate vestitol at the positions 7 and/or 4’; any clear evidence of methylation at 2’ position of vestitol is still lacking [83]. Vestitol is a predominant isoflavonoid produced in *L. japonicus*, present in very small amount in unstressed conditions, but increases significantly at biotic [84,85] or abiotic stresses [62] or after treatment with 10 mM glutathione [78,86]. To a lesser extent, sativan also accumulates in such conditions. Other isoflavonoids, such as formononetin and biochanin A, were raised after UV-B irradiation but their levels remained more than ten-times lower in comparison to vestitol. Accumulation of sativan and medicarpin was also detected, but in an even lower extent [42].

Glycosylation is a major decorative modification that occurs frequently as a last step of the biosynthesis of certain flavonoids or isoflavonoids. UDP sugar residues are attached to the flavonoid core via a uridine diphosphate glycosyltransferase (UGT) [87]. A large number of putative UGT genes have been identified in several plant species. However, only few of them were functionally characterized, mostly in *Arabidopsis thaliana* [88]. In the *L. japonicus* genome, 188 putative UGT genes were identified by genome-wide searching [89]. Tree UGT proteins of the UGT72 family enzymes (UGT72AD1, UGT72AH1 and UGT72Z2) showed narrow substrate preferences to flavonol aglycones in vitro and the overexpression of UGT72AD1 and UGT72Z2 led to increase of flavonol rhamnosides. Another two proteins, UGT72AF1 and UGT72V3, exhibited a broad activity towards flavonoids and isoflavonoids [89]. Such a broad activity of UGTs is known also from other legumes, in particular in the case of four UGTs (GT22D, GT22E09, GT29C and GT29H) from *M. truncatula* [90] and three UGTs (UGT73F2, UGT73C20 and UGT88E19) from *G. max* [91,92]. The UGT activity resulted to high diversity of glycosides in *L. japonicus*; particularly (25) kaempferol and (12) quercetine glycosides were found mostly in flowers [64]. A list of genes related to flavonoid and isoflavonoid pathways in *Lotus japonicus* is shown in Table S2.

## 3. Differential Regulation of Flavonoid and Isoflavonoid Biosynthetic Pathways in *Lotus japonicus*

**Figure 2:**
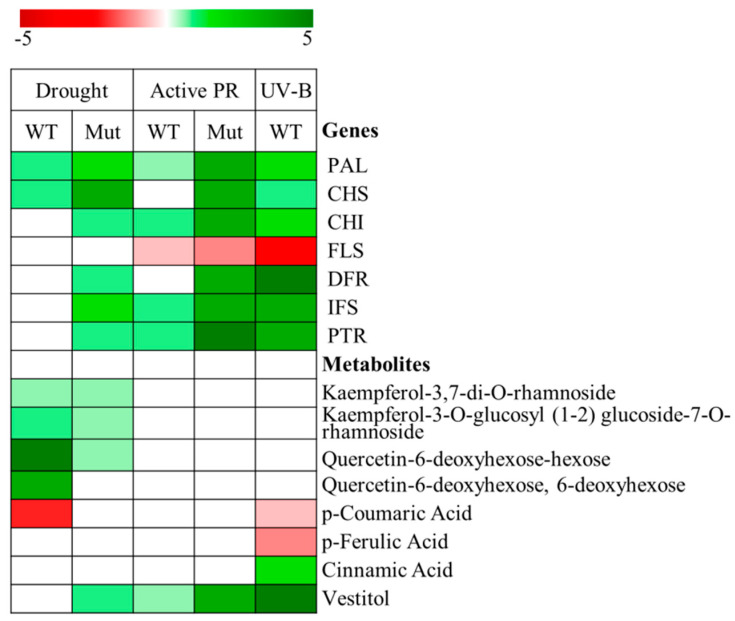
Comparison of the changes in the relative expression levels of the most highly modulated genes (determined by RT-qPCR) and in the levels of metabolites in *Lotus japonicus* leaves, in response to different abiotic stress situations studied (drought, impairment of photorespiration, UV-B irradiation). Changes are indicated as log_2_ of the fold change in gene expression levels and metabolite content for each genotype and stress condition analyzed, relative to the unstressed control plants, as described in previous works [42,62]. Red and green indicate lower and higher levels than in the controls, respectively. The color intensity represents the log_2_ of fold change as indicated in the scale bar. WT, wild type; MUT, *Ljgln2-2* photorespiratory mutant plants deficient in plastidic glutamine synthetase (GS2); active PR, active photorespiration. Active photorespiration is particularly stressful for the *Ljgln2-2* mutant plants due to the high accumulation of ammonium produced as a consequence of the deficiency in photorespiratory ammonium re-assimilation (the impairment of photorespiration) [95,98]. Abbreviations used for genes are described in Figure 1. Given the high number of genes that encode for the flavonoid and isoflavonoid biosynthetic enzymes and the high gene redundancy, a set of specific oligonucleotides were utilized that amplified only specific copies of the redundant gene probesets for key enzymes of the pathways [62]. The results obtained for the most representative and highly expressed gene from each gene family are shown. Genes indicated have the following accession numbers (using Kazusa 3.0 terminology and when available GeneBank codes): PAL, Lj1g3v4590760 (BAF36971.1); CHS, Lj2g3v2124310; CHI, Lj5g3v2288880 (Q8H0G2.1); FLS, Lj1g3v0705350; DFR, Lj5g3v0108500 (BAE19948.1); IFS, Lj4g3v0485090 (BAF64284.1); PTR, Lj3g3v3360890 (BAF34841.1). Semi-systematic names and chemical structures of the indicated metabolites are shown in Table S1.

The biosynthesis of flavonoids in relation to different stresses in plants has been studied by several authors (see [93] as an example). A recent work has established that there is a differential regulation of flavonoid and isoflavonoid biosynthetic pathways in *L. japonicus* in relation to nitrogen metabolism and in response to different stress conditions [62]. An increase in the level of expression of several genes of the isoflavonoid pathway was observed in response to drought or active photorespiration, which was much more noticeable in a *Ljgln2-2* photorespiratory mutant lacking the plastidic isoform of glutamine synthetase (GS2) [1,62] (Figure 2). Therefore, important changes in phenolic metabolism as a result of GS2 deficiency were observed in *L. japonicus* plants in response to stress. The plastidic GS2 isoform is the main point of connection between nitrogen assimilation and photorespiratory metabolism. Both photorespiration and phenylpropanoid metabolism are mainly related to carbon metabolism, but also to nitrogen metabolism because of the use of amino acids as precursors. Therefore, the use of photorespiratory mutants allowed us to understand the cross-interaction between carbon and nitrogen metabolisms as well as (iso)flavonoid metabolism [1,94]. Previous works have clearly established how a GS2 defect in nitrogen assimilation affects carbon metabolism in this plant [1,94,95,96]. In addition, different transcription factors were detected that may be important in the carbon/nitrogen (C/N) balance in *L. japonicus* plants [94]. Several works in different plant species have pointed out that the transcription of flavonoid genes is modulated by the plant’s C/N ratio, and that this regulation seems to be mediated by MYB transcription factors [97]. However, despite numerous studies examining the effects of available carbon (C) or nitrogen (N) on flavonoid biosynthesis, the mechanism of C/N interactive effects on flavonoid metabolism is still unclear [97].

Figure 2 also shows that kaempferol and quercetin, the main flavonols detected in *L. japonicus* leaves, tended to accumulate substantially in response to drought stress in wild type plants [62]. Flavonol accumulation may represent the basic defense system in *L. japonicus* against the increased oxidative stress produced by drought or the impairment of photorespiration, as in many other plants, since it is well known that flavonols are probably the most important flavonoids participating in stress response [8], especially those with dihydroxy-substituted B-ring, which have been reported to play an antioxidant role [99]. However, there are also reports on legumes showing that the accumulation of isoflavonoid phytoalexins and the induction of their biosynthesis may also occur in different types of abiotic stresses, such as UV-irradiation, drought or the presence of heavy metals [100,101,102]. Although vestitol is a clear example of a typical phytoalexin, mainly active in the response to pathogen attack [78], it is possible that the abiotic stress situations perceived by *L. japonicus* plants, at least in some cases, may mimic some stages of the signal transduction pathway that is elicited by biotic stress, thus stimulating the biosynthesis of isoflavonoids over flavonols. Moreover, it has also been reported that isoflavonoid biosynthesis could be elicited using reduced glutathione in *L. japonicus* leaves, a treatment associated with phytoalexin production in response to biotic challenges [45,103]. Interestingly, a strong bias is also documented in soybean toward increasing the expression of isoflavonoid biosynthesis concomitant with some down regulation of other flavonoids such as flavonols, anthocyanins and tannins in response to biotic stress induced by *Pseudomonas syringae* [104]. The differential response between WT and mutant plants observed regarding the biosynthesis and accumulation of different branches of flavonoids or isoflavonoids could be related to changes in C/N balance and to the crucial role of GS2 in this balance in *L. japonicus* plants. GS2 may be connected with some type of regulatory network related to phenolic metabolism in this plant [62].

Recent work has also examined the response of *L. japonicus* plants to UV-B irradiation. Intense ultraviolet radiation is an important stress situation that hampers the growth and productivity of the plants. Under this stress condition, an induction of isoflavonoid biosynthesis in *L. japonicus* was observed [42] (Figure 2). However, in this case a substantial increase in isoflavonoid content produced by UV-B was detected in wild type plants even in the absence of the GS2 mutation. Therefore, a peculiar strategy was observed in *L. japonicus* in different types of abiotic stress situations which resulted in an accumulation of isoflavonoids as a possible alternative to accumulation of flavonols as described in other plant species. The possible function of different isoflavonoids in UV-B defense is still unclear. In *Medicago sativa*, UV-B treatment induced the accumulation of several isoflavonoids, although vestitol was not detected [105]. The increase of several genistein and daidzein derivatives, especially malonylgenistin was observed in soybean [47]. Different responses with respect to isoflavonoids can be found as a result of UV-B radiation, depending on the plant species. In non-legume plants, genes involved in flavonoid biosynthesis pathways were highly induced in leaves after UV-B irradiation [106] and accumulation of flavonoid compounds, mainly glycosilated flavonoids, have been described [106,107]. However, in legumes plants, UV-B radiation increases the accumulation of isoflavonoids and the expression of genes involved in isoflavonoid biosynthesis pathways [102,108,109], although [110] reported that the expression of *GmF3H* and *GmFLS* was induced by UV-B irradiation and their expression stimulated the accumulation of flavonols as kaempferol glycones in soybean plants. In *L. japonicus* it has been suggested that vestitol could play an important role after 16 h of UV-B treatment. Vestitol would act as an antioxidant compound since increase in reactive oxygen species has been described as a common response to different abiotic stress conditions [42]. Different works have reported total peroxyl radical-scavenging capacity of flavonoids [111].

The differential expression of flavonoid genes during nodule formation is of particular interest considering the relevance of these compounds for symbiosis formation. Genes involved in phenylpropanoid synthesis were highly expressed in nodule parenchyma and nodule cortex. Phenylpropanoids provide the main building blocks of both suberin and lignin, which function as a physical barrier or mechanical support at the cell wall. Because nodule vascular bundles are developed through the nodule’s inner cortex, where lignin accumulation is necessary, expression of genes in the phenylpropanoid pathway at nodule parenchyma will be responsible for the synthesis of the nodule vascular bundle [112].

It is noteworthy that a set of genes for the flavonoid biosynthesis pathway was highly expressed in nodule parenchyma. Flavonoids are necessary for forming the nodule by inhibition of polar auxin transport at the site of the rhizobia infection, especially in indeterminate nodules such as *Medicago truncatula* and white clover [113,114]. In the nodulation process of the determinate legume, the flavonoid pathway seemed to be activated in at least four different stages: first, in a nitrogen nutrient deficiency condition to release flavonoids as signal compounds [115]; second, shortly after Nod factor perception as defense-related genes [116]; third, during nodule primordia development [117,118]; and, fourth, in mature nodules without known physiological functions [45,118,119]. Some of those flavonoid-related genes were also identified as glutathione responsive genes, and then seemed to mediate a part of vestitol biosynthesis, suggesting a function of flavonoid derivatives as a chemical barrier against other microbes in the nodules. Flavonoids function as a regulator of auxin flow also in determinate nodules [112].

## 4. Co-Expression Analysis of Potential *MYB* Regulatory Genes in *Lotus japonicus*

As mentioned above, isoflavonoid biosynthesis can be stimulated in *L. japonicus* under certain stress conditions, making it very interesting to further analyze the way these processes are regulated and the transcription factors responsible for the regulation of the isoflavonoid metabolic pathway in this plant. This is particularly important because isoflavonoids in the diet have been linked to anticancer and antiaging health benefits that are associated with their phytoestrogenic and antioxidant properties [103]. In addition, in legumes, there is an extra dimension to the regulatory control of phenylpropanoid metabolism because they produce isoflavonoids that serve as phytoalexins and as signaling molecules for nodulation. Consequently, there is an interest in understanding how isoflavonoid metabolism can be engineered in tissues where their high levels might be beneficial [103]. 

Phenylpropanoid metabolism is regulated spatially and temporally during plant development, and different works have previously shown the importance of MYB transcription factors (TF) as regulators in plants as well as the potential of exploiting MYB transcription factors as a mean of modifying phenylpropanoid accumulation [2]. The gene regulation of plant secondary metabolism involves the formation of the MBW complex, which consists of R2R3-MYB proteins, base-helix-loop-helix (bHLH) proteins and WD-repeat (WDR) proteins [2,120]. This multiprotein complex is based on the interaction between MYB group proteins and the bHLH group or on the interaction between different subgroups of bHLH proteins. MYB transcription factors within this complex may act as activators or repressors in plants and confer the specificity in regulating the different pathways. The R2R3 domain of proteins belongs to the largest groups of MYB factors and is responsible for DNA binding, promoter specificity as well as for the interactions with other cofactors [121]. In some cases, for example in *Vitis vinifera*, bHLH and WD40 factors have not been identified, indicating that not all plants in flavonoid biosynthesis need to retain the MYB-bHLH-WD40 regulatory complex [122]. In the control of flavonoid pathway regulation in *A. thaliana*, there are six important genes that encode transcriptional regulators. Specifically, these are TT2 (from the MYB group), TT8 (from the bHLH group), TTG1 (WD type), TT16 (MADs box), TT1 (zinc finger) and the TTG2 gene [123]. In the *L. japonicus* genome over 100 putative MYB and bHLH genes have been distinguished [103]. Three MYB genes LjTT2a (Lj6g3v1201340.1-3), LjTT2b (Lj6g3v1201220.1) and LjTT2c (Lj6g3v1201370.1) are involved in induction of *LjANR* but not *LjLAR*. LjTT2b expression appeared to be limited to the roots, interacting with TT8 and TTG1. LjTT2c was expressed in all organs examined and showed weak transactivity without TT8 and TTG1. LjTT2a was expressed in response to environmental stresses and had the most diversified activation of the *ANR* promoter, with a low specificity of interaction with bHLH, in addition to little requirement for WDR proteins [124]. All 3 TT2s also activated expression of DFR2 and ANS showing the capacity to control PA synthesis. Similarly, the expression of *LjMYB14* correlates with *ANR*, *ANS* and *LAR1* [125]. Another MYB factor, LjPAP1, activated only the *DFR2* and *ANS* promoter with co-expression of LjTT8 and LjTTG1 but not ANR, suggesting that LjPAP1 is specifically responsible for anthocyanin production [126]. The biosynthesis of flavonols is stimulated by the MYB12 factor that induced the expression of CHS6, F3H and FLS [127].

Considering that MYB proteins represent one of the largest plant TF families and are involved in the regulation of multiple processes [2], the search for specific MYB TF that may regulate isoflavonoid biosynthesis in model legumes such as *L. japonicus* is of fundamental importance. Protein structure and expression patterns were found to be informative for determining the function of individual MYB proteins [2,103,128,129,130]. The authors of [103] initiated this type of study in *L. japonicus* by coupling bioinformatics and co-expression analysis in order to identify candidate genes encoding TF involved in the regulation of isoflavonoid biosynthesis in this plant. The authors concluded that several members of different subgroups of R2R3MYB TFs act coordinately to induce the flux to isoflavonoids and/or reduce the flux of metabolites through competing branches of phenylpropanoid metabolism, such as those leading to flavonols and anthocyanins, so that the precursor metabolite pool can be channeled effectively into isoflavonoids. The most likely regulators of isoflavonoid biosynthesis were found to be genes such as *LjMYB14* which enhanced the expression of the general phenylpropanoid metabolism and some of the genes specific for isoflavonoid metabolism [103]. However, the overexpression of *LjMYB14* was not sufficient to induce isoflavonoid accumulation, suggesting that additional TFs are required for the induction of key genes (*IFS, IFR*) in isoflavonoid biosynthesis. A prime candidate for such an additional regulator of isoflavonoid biosynthesis was found to be *LjMYB152*, which showed similar kinetics but much higher transcript levels than *LjMYB14* after elicitation of the induction of expression of isoflavonoid biosynthetic genes [103]. Interestingly, it has been also suggested that the attenuated expression of a complex integrated by MYB and bHLH would allow metabolites from the flavonoid pathway to be diverted to the isoflavonoid biosynthesis in *L. japonicus* [131]. 

**Figure 3:**
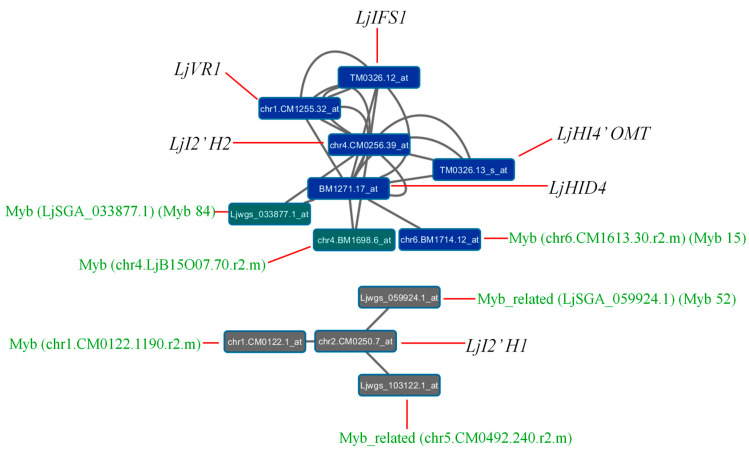
Gene co-expression network developed to analyze the connectivity between isoflavonoid biosynthetic pathway and *MYB* genes. Genes shown with the same color show a similar transcriptional regulation in all data analyzed. Grey lines indicate the interconnections between isoflavonoid and *MYB* genes, and red lines link probesets with gene names or Kazusa codes of genes. The co-expression network was visualized using Cytoscape and analyzed using the NetworkAnalyzer [135]. Gene abbreviations as described in Figure 1 and other codes as reported in Kazusa 2.5 terminology. Gene codes according to the 3.0 version of the *L. japonicus* genome are: *LjIFS1* (Lj4g3v0485090.1); *LjI2’H1* (Lj2g3v1925730.1); *LjI2’H2* (Lj4g3v0189840.1); *LjHID4* (Lj5g3v2057520.1); *LjHI4’OMT* (Lj4g3v0484930.1); *LjVR1* (Lj6g3v0294050.1); LjSGA_033877.1 (Lj4g3v2079370.1); chr4.LjB15O07.70.r2.m (Lj4g3v1787120.1); chr6.CM1613.30.r2.m (Lj6g3v0029920.1); LjSGA_059924.1 (Lj0g3v0337739.1); chr1.CM0122.1190.r2.m (Lj1g3v4830110.1); chr5.CM0492.240.r2.m (Lj5g3v2298100.1).

A recent work aimed to build a gene co-expression network using all the transcriptomic data available for *L. japonicus* in order to analyze the interconnections between nitrogen assimilation and photorespiration [94]. Networks are very useful tools for the prediction of genes co-expressed under different conditions and for the identification of transcription factors that could be involved in the regulation of these genes [132,133,134]. A first co-expression network was constructed using the genes for primary nitrogen assimilation and for photorespiratory metabolism. In addition, a second co-expression network was built using the same type of genes and also different TF genes from *L. japonicus* available in the databases. In that co-expression network, a total of 370 TFs resulted to be connected to at least one gene for nitrogen assimilation and one photorespiratory gene. Some of these TFs could be related to isoflavonoid metabolism considering that both nitrogen and photorespiratory metabolisms are related with isoflavonoid biosynthesis, as mentioned above. An additional gene co-expression network has been set up to establish interconnections between the isoflavonoid biosynthetic pathway and *MYB* and *MYB-*related genes in *L. japonicus* (Figure 3). By using this new gene co-expression network, six different MYB TFs have been identified that could be involved in the regulation of isoflavonoid metabolism in this plant species. Analyzing these results, it was observed that the *LjI2’H1* gene is the only one connected to three MYB genes (LjSGA_059924.1, chr1.CM0122.1190.r2.m and chr5.CM0492.240.r2.m), suggesting that the expression of the *LjI2’H1* gene would be regulated by these TFs. The *LjHID4* gene showed the highest number of connections. This gene was interconnected to other isoflavonoid pathway genes (*LjIFS1*, *LjI2’H2*, *LjHI4’OMT* and *LjVR1*), and to the other three MYB genes identified in the co-expression network: chr6.CM1613.30.r2.m, LjSGA_033877.1 and chr4.LjB15O07.70.r2.m. The other isoflavonoid gene showing connections was *LjI2’H2*. In this case, the *LjI2’H2* gene was interconnected with LjSGA_033877.1 and chr4.LjB15O07.70.r2.m genes. In summary, only *LjI2’H1*, *LjI2’H2* and *LjHID4* genes were interconnected to the MYB genes identified in the co-expression network, suggesting that these six TFs could be involved in the regulation of the expression of these genes which correspond to enzymes that catalyze two consecutive reactions of the isoflavonoid pathway. The other isoflavonoid genes of this route were connected to each other but did not show connections to any of the above-mentioned TFs. Two MYB genes obtained in this co-expression network (LjSGA_033877.1 and chr4.LjB15O07.70.r2.m) were found among 370 TFs connected to genes for nitrogen assimilation and photorespiratory genes [94], confirming again the relation between these two pathways and isoflavonoid biosynthesis. According to the co-expression network presented here, new candidates for isoflavonoid regulation were identified, in addition to the previously proposed ones (*LjMYB14* and *LjMYB152* [103]). Current projects aim to use transposon-tagged LORE1 mutants affected in the genes encoding for MYB transcription factors already identified in *L. japonicus* in order to demonstrate and characterize their possible role in isoflavonoid biosynthesis regulation in this plant.

It is also important to mention here that interconnections between nitrogen availability and (iso)flavonoid metabolism have been clearly shown by different authors, since the content of flavonoids increases in response to nitrogen and phosphorous depletion [136,137,138,139]. Different members of the MYB TF families have been commonly identified as relevant also in these processes. Many other studies have analyzed other aspects of nitrogen fertilization and secondary metabolism (see for example [140,141]). More detailed information and schemes about the intertwining of nitrogen and phenolic metabolism can be found elsewhere [142].

## 5. The Importance of Isoflavonoid Biosynthesis in Soybean: Use of the Knowledge Obtained in the Model Legume *Lotus japonicus* for the Genetic Improvement of Soybean

Particularly important is the transfer of knowledge obtained with the model species *L. japonicus* to a cultivated legume of great economic importance such as soybean. Both legumes have the same type of determinate nodules and genomes with a high degree of synteny [38]. Soybean orthologous genes corresponding to the promising *LjMYB* genes found in *L. japonicus* would be identified and further studied. The positive regulator of soybean isoflavones biosynthesis *GmMYB29* was found to form a cluster with the *L. japonicus* isoflavonoid regulator *LjMYB14* [143]. Mutant collections are being screened in order to develop new commercial soybean varieties [144]. Some positive [143,145,146,147,148,149] or negative [150,151] regulators of isoflavonoid biosynthesis in soybean have already been found; however, there are still many aspects and actors of the regulation of this pathway that have to be discovered. We expect that the advancement produced with the model legume *L. japonicus* will help to make progress in the understanding of isoflavonoid biosynthesis regulation in soybean. This is particularly important considering the beneficial effects of soybean isoflavonoids (isoflavones) on human health, including the prevention of different types of cancer and of cardiovascular diseases [143]. While several efforts are being made in order to obtain soybean varieties with high isoflavonoid content, in some cases a high content of isoflavonoids is not desirable, such as in the case of the soy beans used for infant nutrition formulas, where the estrogenic effect of isoflavonoids may have adverse health effects on the infant development [152]. For these reasons, it is important to obtain new soybean varieties with either increased or decreased content of isoflavonoids, and, in order to do that, it is fundamental to identify all the factors that regulate the biosynthetic pathway of these compounds. In the case of mutants affected in negative regulators, high isoflavonoid content should be observed, while in the case of positive regulators, low isoflavonoid content is expected, and both types of mutants should be of interest. The fact that soybean represents more than 50% of the production of oilseed legumes worldwide [153] together with its importance for human nutrition and health [154,155] make the study of isoflavonoid biosynthesis in soybean of extreme interest. A further interest in the study of MYB mutants in soybean comes from the fact that the knockout of some of these factors can result in increased tolerance to abiotic stresses such as cold [156] or drought [157]. The current authors are actually working on the characterization of specific mutants of the *LjMYB13* and *LjMYB15* genes in order to figure out their possible role in the regulation of isoflavonoid biosynthesis and/or response to stress [79]. The *LjMYB15* gene was interconnected to isoflavonoid pathway genes as described above (Figure 3) and, in a phylogenetic analysis, the *LjMYB13* gene was shown that was in the same clade than *LjMYB15* and other well-known genes that are part of the abiotic stress response in plants [103,143], as well as with genes involved in the regulation of isoflavonoid biosynthesis such as *LjMYB14* and *GmMYB29* [103,143]. A function in isoflavonoid regulation was proposed for *LjMYB15* but not demonstrated [103]. In the case of the *Arabidopsis* homologs of *LjMYB13* and *LjMYB15*, their involvement in the response to drought was demonstrated [158,159]. 

It has been also recently shown that a R2R3-type MYB transcription factor gene from soybean, *GmMYB12*, is involved in flavonoid accumulation and abiotic stress tolerance in transgenic *Arabidopsis* [160]. Interestingly, the authors propose that the higher tolerance to abiotic stress was produced by regulating osmotic balance (increased proline accumulation), together with other factors such as protecting membrane integrity and maintaining ROS homeostasis. Our previous works have also shown that all these factors may be also related with (iso)flavonoid biosynthesis under abiotic stress in a model legume such as *L. japonicus* ([61], and references therein). The research with model legume species improves our knowledge of cultivated legumes, but this is also true vice versa. For example, in a recent report, LjG6DT, the enzyme that catalyzes the prenylation of isoflavonoids in *L. japonicus* [161], was discovered thanks to the knowledge previously obtained about isoflavonoid prenylation in soybean. Studies with both cultivated and model species are not mutually exclusive but rather they complement each other, like in the case of *L. japonicus* and soybean. 

## 6. Conclusions and Future Prospects

This paper summarizes recent advances made in flavonoid and isoflavonoid research in the model legume *L. japonicus*. The study of the response of *L. japonicus* to abiotic stress conditions led to different novel findings, such as the accumulation of new flavonols that were described for the first time in *L. japonicus* leaves [62] and of a peculiar pattern of isoflavonoid accumulation in the response of this plant to UV-B irradiation [42]. Despite the fact that flavonoid and isoflavonoid metabolism is a very active field of research; several aspects of these pathways are far from having been completely described. Technical advances in metabolomics are enabling the discovery of a growing number of flavonoids and isoflavonoids structures. However, chemical modification of the flavonoid/isoflavonoid scaffolds, such as glycosylation and acylation, add another layer of complexity to their chemical diversity; and the reason beyond such complexity is still not completely understood. Legumes also use flavonoids or isoflavonoids in order to attract their chosen symbiont in a species-specific way. Despite the important role played by *L. japonicus* in elucidating the molecular genetics of legume–rhizobia symbiosis, it is still unknown which class of phenolic compounds are used by this species in order to attract its chosen symbiont [15]. Studies of the symbiotic capacity of specific *L. japonicus* mutants impaired in specific branches of the biosynthesis of phenolic compounds, paired with metabolite profiling will be needed in order to fill this gap. The regulation of isoflavonoid metabolism is also far from being completely understood. A few negative and positive regulators have been identified in soybean, while no clear isoflavonoid regulators have been identified in *L. japonicus* to date. The co-expression analysis presented in this paper identified potential candidates for isoflavonoid regulation in *L. japonicus*. Future works should be aimed to the characterization of specific mutants in these genes in order to understand whether they are involved in isoflavonoid regulation, and also if they may play a role in the response to different kinds of abiotic stress. A deeper understanding of isoflavonoid regulation may also permit tackling the genetic improvement of soybean and to breed varieties with either increased or decreased isoflavonoid content, two opposite traits that can be desirable depending on the products that will be manufactured using these soya beans. Since most of the regulators identified in these species are from the MYB transcription factor family, which is composed of a very high number of genes, traditional approaches, such as searching for isoflavonoid-related QTL, may be very time consuming. Bioinformatics approaches, such as the construction and analysis of gene co-expression networks in order to find new candidate regulators, combined with validation of these genes by characterizing loss-of–function mutants, have already showed promising results. Finally, as explained in this review, in order to broaden the knowledge of flavonoid and isoflavonoid metabolism and regulation, studies that take into consideration both model species such as *L. japonicus*, of easier genetic manipulation, and cultivated species of great economic importance, such as soybean, will be of paramount impact for legume flavonoid/isoflavonoid research. 

## Supplementary Materials

The following are available online at https://www.mdpi.com/2223-7747/9/6/774/s1, Table S1: Overview of the names and chemical structures of relevant compounds referred to this review. Table S2: List of genes related to flavonoid and isoflavonoid pathways in *Lotus japonicus*.
