# Supplementary material for: Flavonoids and Isoflavonoids Biosynthesis in the Model Legume Lotus japonicus; Connections to Nitrogen Metabolism and Photorespiration
Source: Plants (Basel). 2020 Jun 20;9(6):774. doi: 10.3390/plants9060774 (PMC7357106; doi:10.3390/plants9060774)
Supplement: Supplementary file 1 [file plants-09-00774-s001.zip › Supplementary Table S1.docx]

Supplementary Table A1: Overview of the names and chemical structures of relevant flavonoids and isoflavonoids in *L. japonicus* referred to this review.

| Trivial (common) name | Semi-systematic name | Chemical structure |
| --- | --- | --- |
| ***Isoflavonoids:*** |  |  |
| - | *2,7,4‘-Trihydroxyisoflavanone* |  |
| - | *2,7-Hydroxy-4‘-O-methoxyisoflavan-4-one* |  |
| - | *7,2'-Dihydroxy-4'-O-methoxyisoflavanol* |  |
| 2-Hydroxyformononetine | *7,2‘-Dihydroxy-4‘-O-methoxyisoflavone* |  |
| Biochanin A | *5,7-Dihydroxy-4'-methoxyisoflavone* |  |
| Formononetine | *7-Hydroxy-4‘-O-methoxyisoflavone* |  |
| Medicarpin | *9-Methoxypterocarpan-3-ol* |  |
| Sativan | *7-Hydroxy-2',4'-dimethoxyisoflavan* |  |
| Vestitol | *2,7-Dihydroxy-4'-methoxyisoflavan* |  |
| Vestitone | *7,2'-Dihydroxy-4'-O-methoxyisoflavan-4-on* |  |
| ***Flavonoids:*** |  |  |
| Catechin | ***(+)-(2R:3S)-5,7,3',4'-Tetrahydroxyflavan-3-ol*** |  |
| Cyanidine | ***3,3',4',5,7-Pentahydroxy flavylium*** |  |
| (-)-Epicatechin | ***(-)-3 3' 4' 5 7-Tetrahydroxyflavan-3-ol*** |  |
| Isoliquiritigenin | *4,2',4'-Trihydroxychalcone* |  |
| Kaempferol | *3,5,7,3'-Tetrahydroxyflavone* |  |
| Kaempferol-3,7-di-O-rhamnoside | *3,5,7,3'-Tetrahydroxyflavone-3-O-β-glucopyranoside-7-O-rhamnopyranoside* |  |
| Kaempferol-3-O-glucosyl-(1→2)-glucoside-7-O-rhamnoside | *3,5,7,3'-Tetrahydroxyflavone-3-O-β-glucopyranosyl-(1→2)-β-glucopyranoside-7-O-rhamnopyranoside* |  |
| Liquiritigenin | *4',7-Dihydroxyflavan-4-one* |  |
| Naringenin | *4’,5,7-trihydroxyflavan-4-one* |  |
| Naringenin chalcone | *2',4,4',6'-Tetrahydroxychalcone* |  |
| Quercetin | *3,5,7,3',4'-Pentahydroxyflavone* |  |
| Isorhamnetin | *3,5,7, 4'-Tetrahydroxy-3'-methoxyflavone* |  |
| Gossypetin | *3,5,7,8,3',4'-Hexahydroxyflavone* |  |
| **Simple phenolics:** |  |  |
| p-Coumaric Acid | *4-Hydroxycinnamic acid* |  |
| p-Ferulic Acid | *4-Hydroxy-3-methoxycinnamic acid* |  |
| Cinnamic Acid | *trans-3-Phenylacrylic acid* |  |
